# Epitope Imprinted Nanoparticles as Customized Synthetic Antibodies for Biorecognition in Theranostics

**DOI:** 10.1007/s40883-025-00478-x

**Published:** 2025-12-05

**Authors:** Simão P. B. Teixeira, Rui L. Reis, Rui M. A. Domingues

**Affiliations:** 1 3B’s Research Group, I3Bs – Research Institute on Biomaterials, Biodegradables and Biomimetics, Headquarters of the European Institute of Excellence On Tissue Engineering and Regenerative Medicine, University of Minho, AvePark – Parque de Ciência e Tecnologia, Rua Ave 1, Edifício 1 (Sede), Barco, Guimarães, 4805-694 Portugal; 2 ICVS/3B’s–PT Government Associate Laboratory, Braga/Guimarães, Portugal; 3 INL – International Iberian Nanotechnology Laboratory, Avenida Mestre José Veiga S/N, 4715-330 Braga, Portugal

**Keywords:** Biomarkers, Epitope imprinting, Molecular recognition, Nanoparticles, Targeted therapies, Theranostics

## Abstract

**Abstract:**

As medicine advances towards an era of personalized and targeted therapies, the need for precise and reliable molecular recognition elements becomes increasingly critical. These elements are essential for directing therapeutic agents to specific cells or tissues, thereby maximizing efficacy while minimizing off-target effects. Among the emerging technologies, epitope-imprinted nanoparticles (EINPs) have demonstrated exceptional potential as “synthetic antibodies” for the recognition of biomarkers. These nanoparticles are designed to mimic the binding capabilities of natural antibodies but with enhanced stability, specificity, and ease of production. Unlike traditional antibodies, which can be costly, labile, and prone to significant side effects, EINPs offer a cost-effective, robust, and customizable platform for targeted sensing and therapeutic applications. This Perspective Review explores the design, synthesis, and application of epitope-imprinted nanoparticles, highlighting their advantages and potential to revolutionize the landscape of abiotic molecular recognition in disease detection and targeted therapies. Finally, we present perspectives on foreseeable breakthroughs and advances, offering insights into how molecular imprinting techniques can be expanded to new biomedical fields, such as tissue engineering.

**Lay Summary:**

Personalized medicine requires precise tools to identify and target diseased cells while avoiding off target effects. Epitope-imprinted nanoparticles (EINPs) are emerging as synthetic alternatives to antibodies, designed to recognize disease markers with high stability, low cost, and fewer limitations than natural antibodies. By mimicking biological recognition, EINPs show promise in applications such as disease detection, drug delivery, and tissue engineering. This perspective review focus on how these nanoparticles are created and applied, and highlights their potential to transform regenerative medicine and targeted therapies through more reliable, customizable molecular recognition systems.

## Introduction

As society evolves progressively into an era of precision medicine, targeted therapeutic and diagnostic approaches have been acquiring a more central role in order to enhance the specificity and efficacy of medical treatments and disease prevention [1]. Many of these strategies rely on the molecular recognition of cell surface *biomarkers*—molecules expressed on the outside of cell membranes that are unique to certain cell types or disease states. Biomarkers are crucial for identifying diseased cells, such as cancer cells, allowing for accurate profiling of disease phenotypes, precise drug delivery, and real-time monitoring of treatment effects. It is worth noting that, in the context of targeted therapies, these cell surface molecules are typically designated as *targets*. By exploiting these molecules, tailor-made theranostics tools can selectively deliver therapeutic agents to target cells while sparing healthy tissues, thus reducing undesired side effects and improving treatment outcomes. This approach not only enhances the efficacy of treatments but also allows for personalized interventions tailored to the unique molecular profile of each patient. Molecular recognition of cell markers is also seen as essential for the deployment of effective biomaterials for tissue regeneration, promoting selective cell adhesion, proliferation, and differentiation, as well as allowing the fabrication of functional scaffolds capable of triggering or responding to specific cellular cues [2].

Over the past few decades, biomedical strategies involving molecular targeting have leveraged the use of antibodies, particularly monoclonal antibodies (mAbs) owing to their high affinity and selectivity toward biomolecules of interest [3]. However, their lengthy and expensive production processes [4], short shelf-lives and half-lives (pre- and post-administration, respectively), and significant immunogenic risks [5] make them suboptimal tools. These limitations have generated interest in the development of alternative binding partners with a similarly specific molecular recognition capability, for example, peptides or aptamers [5, 6]. These molecules present several advantages over traditional antibodies, such as easier chemical modification, predicable structures, programmability, automated synthesis, and higher stability. Yet, their nature as small biomolecules also makes them prone to fast clearance and degradation. Perhaps more importantly, their discovery and development procedures are long, costly, and somewhat inefficient, making them impractical for a large number of research applications and laboratories [7, 8]. Therefore, there is a pressing need for alternatives, which have similar molecular recognition capabilities, but are simultaneously stable, scalable, and cost-effective, rendering them more widely accessible [9].

In this context, molecular imprinting (MI) has been increasingly demonstrating its potential to generate fully synthetic materials with abiotic affinity for biomolecules aiming at various biological applications [10, 11]. Synthetic polymers that can be designed to recognize and bind biologically relevant molecules are of great importance and influence a number of emerging technologies [9], either as single systems or incorporated into other platforms that can assist in the identification of particular compounds or cell markers related with a number of diseases, for example, specific cancer types [12] or autoimmune conditions [13]. The potential presented by the recognition of these molecules by polymeric systems is significant for both diagnostic (e.g., bioimaging) and therapeutic purposes, which can be further combined into integrated theranostics platforms [14, 15].

This Perspective Review covers essential aspects of MI for biomolecular targets using the epitope template approach, mainly focusing on key parameters of epitope selection and design, and some of the latest applications that show the efficacy and versatility of this technology. Moreover, it offers prospects for future developments based on MI polymers in the biomedical field, particularly foreseeing its large-scale adoption for regenerative medicine strategies.

## The Concept of Molecular Imprinting

MI is a technique used to create specific recognition sites within polymer networks by crosslinking monomer(s) in the presence of a template molecule, which corresponds to the recognition target [16]. During this process, a monomer solution is mixed with the template, forming temporary interactions between them. After crosslinking and polymerization, the template is removed, leaving behind a polymer structure with cavities that are complementary to the shape and chemical structure of the imprinted molecule. Importantly, these nanocavities retain the precise spatial organization of molecular interactions necessary for recognizing the target. As result, molecularly imprinted polymers (MIPs) can selectively recognize and bind to their targets through a “lock and key” mechanism similar to those found in natural systems like antibodies and enzymes. Thus, these biomimetic polymer networks should be created by carefully designing the interactions between the network’s building blocks and the specific ligand in order to maximize the recognition potential.

MIPs have garnered increasing attention due to several advantages, including their high affinity and selectivity, which enable them to rival natural biomolecule analogs such as antibodies [11], while simultaneously exhibiting greater chemical stability over time and under various environmental conditions. These properties allow for prolonged transport and storage without cold chains [17] and increase the expected function and circulation time in the human organism. Moreover, their production is comparatively simpler, faster, and less costly, making them more widely accessible to a wider variety of applications [18]. These characteristics, along with the tailor-made construction allowed by a large library of available building blocks, make MIPs particularly suitable for the generation of artificial receptors for biomolecules with no natural binding partners or with incompletely solved structures [7, 19].

Although synthetic recognition of small compounds is now standard practice, MI of proteins traditionally presented significant challenges due to their environmental sensitivity and chemical incompatibility [20]. This required the development of new bulk imprinting methods using water-soluble monomers, essentially based on acrylate and acrylamide derivatives [21] that resulted in materials essentially behaving like hydrogels [14]. However, the production of densely crosslinked polymer structures was necessary in order to ensure the retention of effective binding sites in these networks, greatly hindering protein rebinding since their diameter is often larger than the hydrogel mesh sizes. Additionally, common washing methods for template removal could compromise the integrity of imprinted sites, also contributing to a lower imprinting efficiency. These limitations have been, to a large extent, smartly bypassed by the emergence of surface imprinting techniques, in which MI occurs only at the surface of thin polymer layers, instead of in bulk [22, 23]. This not only allows free template diffusion in solution but also potentially reduces the amounts needed for imprinting procedures.

Despite these developments, other relevant limitations of direct biomacromolecule imprinting remain unsolved. For example, the predominance of strong hydrogen bonds with water often disrupts template-monomer interactions, thus decreasing imprinting effectiveness [24]. This presents a particularly high barrier when targeting molecules rich in hydroxyl and other strongly hydrophilic groups, particularly saccharides. Moreover, the structural complexity of proteins further contributes to these problems, adding an extra layer of difficulty to their MI [25]. The large number of functional groups spread over different regions of protein structures, along with their conformational flexibility, strongly contribute to producing poorly fit binding sites for later recognition. Lastly, but also critically, is the prohibitive cost of most biologically relevant proteins (e.g., growth factors, cellular receptors), which makes them unreasonable options as imprinting templates in large scale [14, 26].

### Epitope-Imprinting Emerges as Solution

**Fig. 1:**
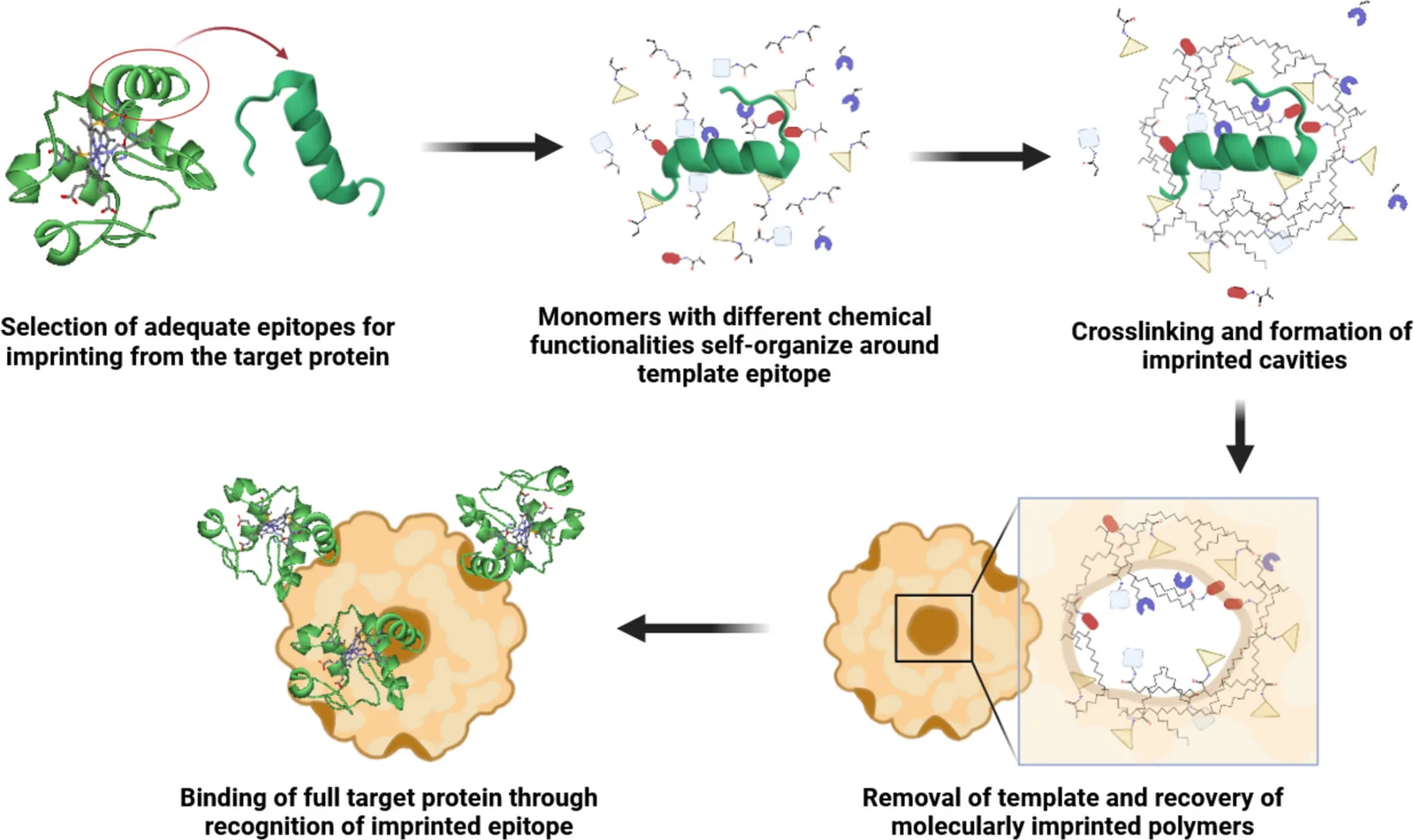
The concept of epitope imprinting implies the recognition of a macromolecule through the imprinting of a small domain selected from its full chemical structure—the epitope

The described list of limitations made biomacromolecule imprinting an inefficient concept, setting back the development of materials for synthetic recognition of proteins until the proposal of *the epitope imprinting approach* [27]. This strategy is inspired by the functioning mechanisms of natural antibodies, which recognize only a small, exposed domain out of a larger molecule, termed an epitope [25]. Similarly, this imprinting approach consists in selecting a portion of the target molecule, which is used as the template for MI instead of the full macromolecule. After its removal, the resulting MIP can rebind the full target by specifically recognizing this particular segment (Fig. 1). While initially explored for the recognition of small oligopeptides [28, 29], it did not take long before the first applications targeting full proteins were reported [30, 31]. Not long after, the concept of epitope-imprinted nanoparticles (EINPs) as *plastic antibodies* was introduced, showing that such materials could compete in functionality and effectiveness with their biological counterparts, having their bioactive potential demonstrated for in vitro cell culture [32] and in vivo as antitoxins [33].

Opting for smaller compounds as templates helps bypass the structural and financial challenges typically associated with larger molecules while still preserving selective recognition and affinity for the larger targets. Their reduced size results in less intramolecular flexibility and fewer functional groups, which contributes to more uniform and consistent imprinted cavities. Unlike proteins, small peptides do not denature because they lack complex tertiary and quaternary structures, and they can also be synthesized to dissolve in a variety of solvents beyond water, allowing their use in different MIP synthesis conditions [25]. However, even though smaller epitopes reduce diffusion-related issues during template removal, their bulk imprinting for later full protein recognition still faces limitations because polymer mesh size hinders subsequent diffusion and rebinding of the targeted larger macromolecules. As a result, epitope imprinting is particularly advantageous for strategies involving surface imprinting, for example, on nanoparticles, where the binding sites are more readily accessible [28]. Additionally, smaller templates are more cost-effective and easier to produce through chemical synthesis [34], allowing the recognition of biologically relevant molecules at much lower costs.

Due to these impactful imprinting concept improvements, research into protein MI has grown exponentially, showing promise for a wide array of applications, including biosensing [35], separation technology [36], targeted drug delivery [37], or tissue engineering [38]. This has prompted a number of excellent reviews on the topic, covering relevant points regarding MI synthesis methods, standardized evaluation parameters, and applicability [11, 14, 20, 35, 39, 40], including our own, in which we have comprehensively reviewed the most crucial features of epitope imprinting technology [22]. Here, we provide a brief overview and update on the basic but relevant concepts of epitope imprinting, with particular focus on the crucial element of epitope design, contextualized by concentrating on its application for the development of systems recognizing cell surface biomarkers and targets for theranostics purposes. It covers the most recent works on this field, highlighting key breakthroughs and presenting a critical perspective on our vison for future research directions.

## Looking for the Right Epitope

**Fig. 2:**
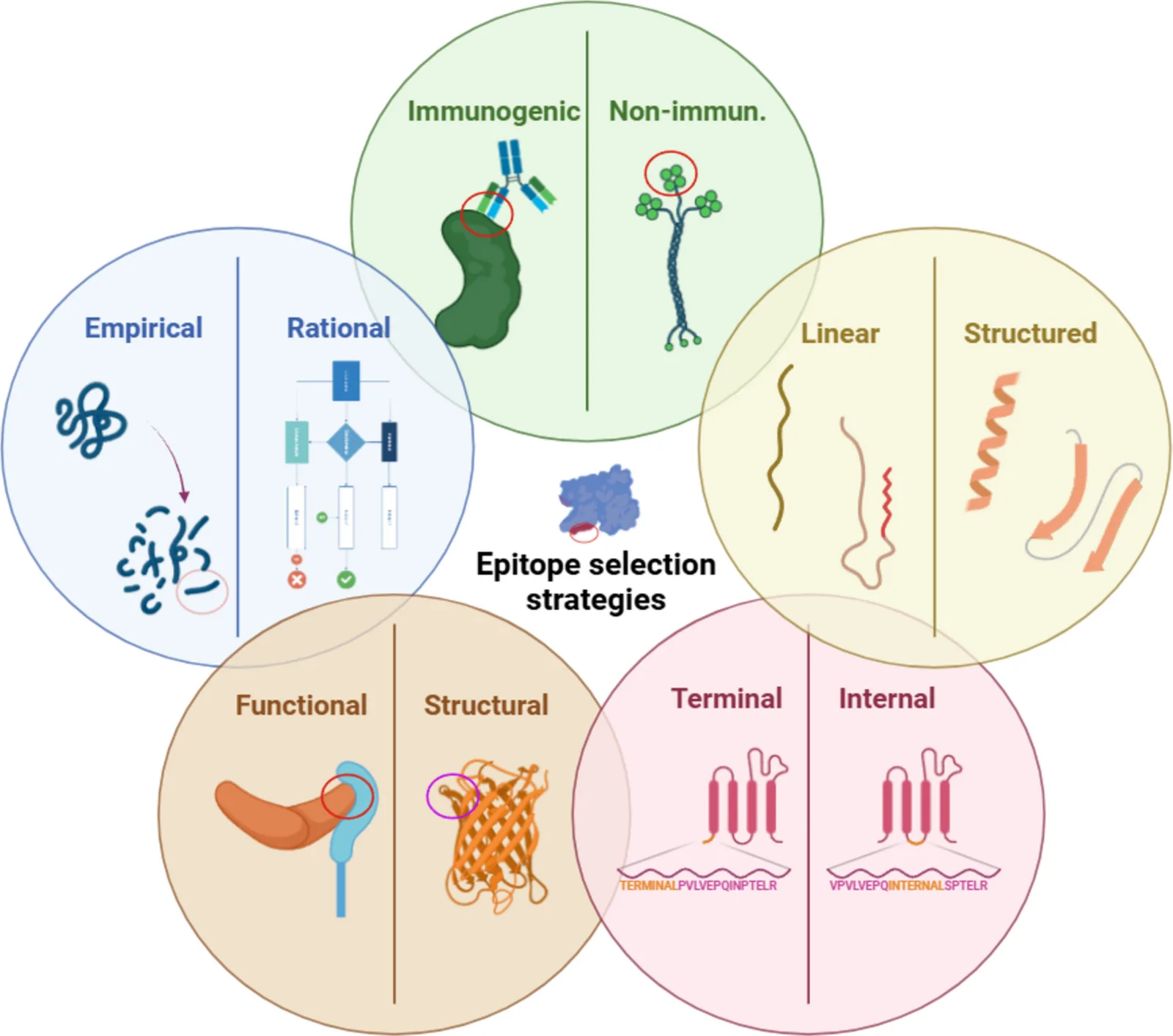
Overview of the different strategies for selection of epitopes for molecular imprinting

Arguably, the most important step for the success of each new epitope imprinting process is the epitope selection. Replacing the full target biomolecule with a smaller alternative template requires an adequate selection procedure to ensure a good correspondence between the imprinted cavities and the final target. This means that the epitope must correspond to a monomer sequence unique to the parent macromolecule, while simultaneously being suitable for the MI synthesis procedures and located in an accessible region in its native form for later rebinding. Recent progress in the field has led to increased interest in saccharide MI, aiming to identify specific glycoproteins [41, 42] or aberrant glycosylation sites on cell surfaces [43, 44], often linked to particular illnesses. Nonetheless, peptides remain largely the most common type of epitope used in MI protocols. Figure 2 provides a concise overview of the available strategies for this selection. Briefly, these strategies can be categorized into empirical vs. rational; terminal vs. internal; immunogenic/functional vs. structural; linear vs. structured.

### Empirical Selection Processes

#### Terminal Protein Sequences

The most straightforward and traditionally used empirical method for epitope selection is the use of a linear peptide corresponding to the N- or C-terminal sequence of the target protein [27, 31]. The main advantages are (i) since these sites are not commonly targeted for post-translational modifications, the template peptide is very likely to directly correspond to the target protein portion, and (ii) these sequences are frequently exposed on protein surfaces, thereby increasing the probability of target recognition by EINPs [45]. Moreover, this selection can be quickly performed by simply looking at the target protein sequence in commonly accessible databases [46], such as UniProt or RCSB protein databank (PDB) [47–49]. Using linear peptides as MI templates is also a less expensive option compared to conformational ones, avoiding the complexity associated with three-dimensional structures, while also facilitating modification or conjugation reactions that increase the peptide functionality (e.g., oriented immobilization) [50, 51]. In a remarkable example of this strategy, Canfarotta et al. prepared dual-imprinted nanoparticles for drug delivery to specific cancer cells, by simultaneously imprinting them against the cytotoxic drug doxorubicin and the C-terminal linear sequence (SLNITSLGLRSLKEISDG) of the extracellular portion of the epidermal growth factor receptor (EGFR), which is exposed at the protein surface. A cysteine residue was added to the epitope to allow its oriented immobilization on the solid phase used in the MI synthesis procedure. The prepared EINPs attained an equilibrium dissociation constant (*K*_*D*_) of 3.6 nM for the binding to the extracellular domain of the EGFR protein and were able to selectively recognize and deliver doxorubicin to cells overexpressing this surface target.

The use of N-termini is not as frequent as C-termini, mostly because the N-terminus may be hydrolyzed or truncated due to signal peptide removal [52]. Nevertheless, Qin et al. showed the efficacy of this approach by producing biodegradable MIP nanocapsules against the N-terminal epitope of CD59 glycoprotein (YNCPNPTADCK) [53]. Using *N,N*′-diacrylylcystamine as the cross-linker, these structures were selectively degraded in the more acidic tumor microenvironment, leading to the release of encapsulated doxorubicin and the selective killing of MCF-7 cells (which overexpress CD59). Additionally, by embedding carbon quantum dots in MIPs, they also functioned as bioimaging tools, thereby demonstrating the theranostic potential of EINPs.

The Z. Liu group has been particularly active in exploring this approach, demonstrating its versatility in combination with multiple synthesis routes. For example, the N-terminal nonpeptide of transmembrane glycoprotein NMB (GPNMB), a marker of triple negative breast cancer (TNBC), was used as template to imprint silica-based nanoparticles by a novel method termed reverse microemulsion-confined epitope-oriented surface imprinting and cladding (ROSIC). These EINPs demonstrated simultaneously impressive selectivity and affinity for the target protein, allowing in vivo targeted imaging of TNBC-bearing mice using GPNMB-specific NIR797-doped nanoparticles [54]. EINPs targeting this protein were further redesigned to work as “nanobeacons,” displaying numerous haptens that helped to recruit endogenous antibodies, thereby effectively directing innate immune mechanisms towards GPNMD-positive tumors in vivo, constituting a promising targeted cancer immunotherapy [55].

**Fig. 3:**
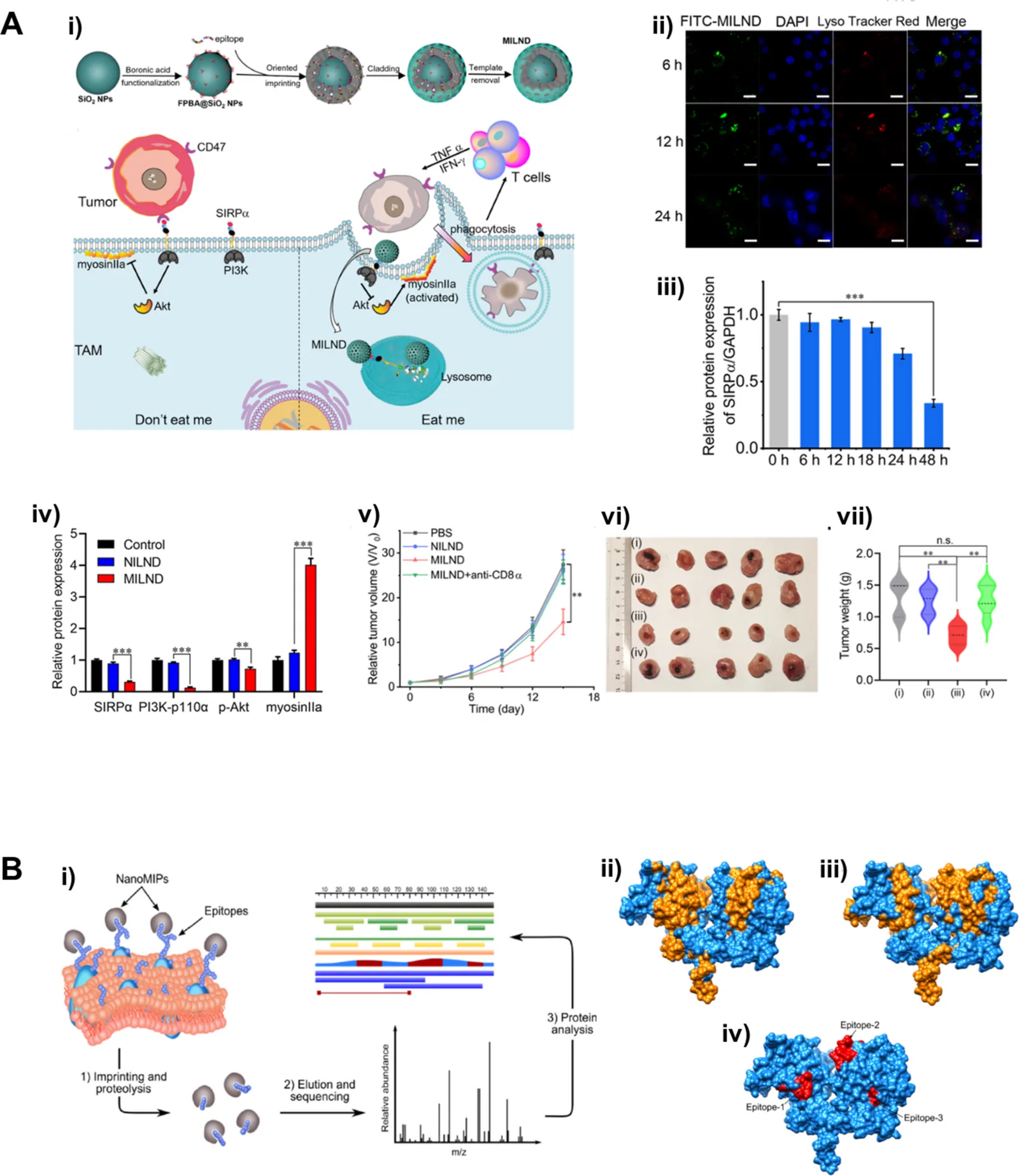
Empirical epitope selection methods. **A** Imprinting of N-terminal epitope of tumor surface target SIRPα for anti-cancer immunotherapy. (i) Illustration of the preparation route of molecularly imprinted lysosomal nanodegrader (MILND) and immunotherapy approach by MILND via blocking the SIRPα-CD47 signaling pathway. (ii) Fluorescence images demonstrating the uptake of MILND by tumor-associated macrophages (green: fluorescein-labelled MILND, red: lysosomes stained by Lyso Tracker Red, blue: DAPI-stained nuclei). (iii) Relative protein expression of MILND-targeted-SIRPα protein degradation in macrophages measured by western blot. (iv) Relative protein expression of SIRPα, phosphatidylinositol 3-kinase, catalytic subunit alpha (PI3K-p110α), phosphorylated protein kinase B (p-Akt) and myosin IIa in control, MILND, or non-imprinted NILND treated cells. (v) Relative tumor volume growth curves of CT26 syngenetic tumor mice over 15 days treatment. (vi) Photograph of the dissected tumors from the CT26 syngenetic tumor mice after 15 days of therapy in each treated group. (vii) Average tumor weight resected from the CT26 syngenetic tumor mice after different treatment. Adapted with permission [56]. Copyright 2024, Elsevier. **B** Advanced empirical epitope selection by *snapshot imprinting*. (i) Diagram depicting the snapshot imprinting process, wherein cell surface proteins are imprinted, the resultant EINPs are collected, and the template peptides eluted and sequenced in order to identify novel biomarkers. (ii–iv) Extracellular region of epidermal growth factor receptor (EGFR); regions considered to be epitopes highlighted in orange, remainder in blue. Epitopes selected from: (ii) label-free deep proteome analysis; iii) snapshot imprinting. (iv) Depiction of epitopes ultimately selected for imprinting highlighted in red. Adapted under CC BY 4.0 [12]

These authors also developed a remarkable anti-cancer therapeutic strategy based on EINPs termed “molecularly imprinted lysosomal nanodegraders” (MILND), using as templates the N-terminal epitopes of different cancer-associated immunosuppressor proteins, namely, signal regulatory protein alpha (SIRPα) and programmed death–ligand 1 (PD-L1) [56, 57]. These EINPs could specifically bind the target protein, either SIRPα on tumor-associated macrophages or PD-L1 on cancer cells, signaling these molecules for lysosomal degradation, thereby reversing the immunosuppressing CD47-SIRPα or programmed cell death protein 1 (PD-1)/PD-L1 signaling and restoring the recognition capability of macrophages and T cells, respectively, towards cancer cells. This resulted in substantial tumor growth inhibition in tumor-bearing mouse models, proving the effectiveness of this technology for improved immunotherapy (Fig. 3A). The same group also synthesized EINPs against the N-terminus of vascular endothelial growth factor (VEGF), which allowed these particles to recognize the two major pro-angiogenic isoforms of this growth factor (VEGF_165_ and VEGF_121_) with high affinity [58]. These synthetic nanomedicines could thus block VEGF signaling in the cancer microenvironment, showing a potent anti-angiogenic effect that led to tumor growth suppression. Both strategies constitute major improvements compared to current anti-cancer therapies, demonstrating great promise for drug-free approaches that can achieve effective tumor suppression with few to no side effects.

Altogether, several studies have concluded that neither N- nor C-termini have proven to lead to inherently superior imprinting performance compared to one another [59, 60]. The choice for each particular target appears to be more complex and dependent on several physicochemical criteria, such as solubility in adequate solvents (porogens), compatibility with imprinting method, or peptide length. The length of the template sequence is particularly crucial, being Limited in two ways. On the one Hand, peptides shorter than 8 amino acids (a.a.) hardly function as idiotypic, with a higher likelihood of corresponding to multiple protein targets [31]. This is why a large portion of epitope imprinting works use 9-mer templates, considered to represent a near-unique code in unstructured domains for the identification of specific proteins. On the other Hand, sequences beyond 16 a.a. can become too flexible and undergo intramolecular interactions, thus acquiring three-dimensional conformations different from the native protein that reduce the imprinting efficiency [61]. Between these limits, research data has shown that longer sequences generally lead to increased specificity and better recognition by EINPs, by providing more molecular interactions between the imprinted nanoparticle and the target. But, interestingly, the molecular weight of the epitope seems to correlate more strongly with the resulting EINP affinity than its a.a. number [62]. This was exemplified by Tai et al., who demonstrated that a heavier 9-mer peptide generated EINPs with a *K*_*D*_ of 10 nM, compared to 20 nM using a Lighter 10-mer peptide. Probably, heavier peptides tend to retain a more stable conformation during polymerization, resulting in more uniform imprinted cavities. Moreover, the compared affinities of each MIP reveal differences when tested against the epitope template vs. against the full target protein. Thus, we can conclude that there is no ideal template peptide length within the 8–16 a.a. range and that it is therefore important to analyze the success of imprinting experimentally with the whole protein in an ad hoc manner.

#### Advanced Experimental Selection Methods

Considering that terminal sequences are not always the best epitope for each protein, more complex methodologies started being adopted to select adequate imprinting templates. One of those methods involves the construction of a.a. sequences with certain physicochemical properties of interest for a particular MI protocol or application. For example, in a study with the goal of detecting natriuretic peptide hormones in blood samples, 9-mer peptide epitopes were chosen based on two premises: possessing an isoelectric point (pI) close to the target molecule and boasting a balanced ratio between hydrophobic and hydrophilic residues [63]. This simple method helped to generate MIPs with impressive dissociation constants (2–20 pM), as well as low reported cross-reactivity. In other works, aiming to detect Regenerating Protein 1B, a biomarker of Pancreatic ductal adenocarcinoma, researchers scanned the target protein sequence and selected seven oligopeptides from different subregions considering, among other properties, their solubility in dimethyl sulfoxide. In one of the reports, the best performing peptide owed its success to the good solubility provided by 6 charged (EDREDD) and 3 polar (NTY) residues [64]. These results demonstrate that considering the physicochemical properties of epitope templates is a good practice during their selection to heighten the performance of produced EINPs. Nonetheless, in their second work, the authors show that one of the chosen peptides improved sensor accuracy probably because of its better exposure at the protein surface compared to the other epitopes [61]. Thus, a more rational epitope selection method that also accounts for the structure of the full protein can likely improve the quality of synthesized MIPs, reducing the time and costs associated with testing numerous alternative templates.

In alternative, Bagán et al. have reported a promising experimental method to directly derive surface-accessible epitopes from the target protein. This involves the immobilization of the protein on a solid phase support, namely, silica nanoparticles, followed by its enzymatic digestion. After washing away the digested portions, only the surface-exposed peptides, which were covalently bound to nanoparticle supports remained. These surface-immobilized epitopes could then be used to produce MIPs, which were able to recognize the target protein [46]. The resulting MIPs can be compared to polyclonal antibodies, with affinity towards an undetermined variety of epitopes. This approach significantly reduces the development time, bypassing the epitope selection step while ensuring the imprinting of surface-accessible peptides. On the downside, it requires the use of full proteins during the synthesis process, nullifying some of the advantages sought by epitope imprinting, particularly concerning costs of implementation. Additionally, the MIPs prepared by this method demonstrated a relatively unsatisfactory degree of selectivity, indicating the need for further experimental refinement before considering a widespread use of this protocol.

In a comparable development, boronate-affinity glycan-oriented surface imprinting represents a step forward for this strategy [65]. In this innovation, while the rationale remains similar, glycans are imprinted instead of peptides. The working principles of this concept restrict its use to glycated proteins, but these correspond to more than 50% of the total proteins in mammalians, representing a large portion of extracellular membrane-associated proteins [66]. Moreover, glycoproteins are often used as disease biomarkers and therapeutic targets [67]. This strategy relies on the reversible covalent interactions that boronic acids can establish with cis-diol-containing molecules, such as sugars. In alkaline conditions, these species form stable cyclic esters, dissociating when the pH switches to acidic values. Thus, this ability can be leveraged for facile template immobilization and removal post-imprinting. The studies which first pioneered this concept achieved MIPs with remarkable selectivity, particularly when compared to the previous peptide-imprinting approach [41, 68], demonstrating the potential of this technique. On the other hand, the affinity displayed for the target saccharide is low compared to other imprinting approaches (*K*_*D*_ in the micromolar range), although of similar magnitude to natural sugar-binding molecules such as lectins. Further refinements (e.g., monomer mixture) might improve the quality of MIPs prepared by this technique.

Alternatively, target protein digestion can be applied as a first step to identify and isolate proteotypic peptides for subsequent imprinting procedures [69, 70], having been employed, for example, on the precise identification of signature peptides of pro-gastrin-releasing peptide in serum samples. More recently, the Piletsky group introduced an ingenious advance in this approach [12, 71, 72], which was termed “snapshot imprinting” (Fig. 3B). In this technique, MIPs are first synthesized in the presence of the target protein(s), generating “protein-MIP complexes,” with non-reacted and low-affinity material being washed out of the mixture. Then, these complexes undergo enzymatic or chemical digestion, where non-bound peptides are also washed. In this manner, only the peptides corresponding to the imprinted cavities remain bound to the polymer complex. Lastly, these peptides are separated and sequenced, allowing the identification of surface-exposed portions of the target protein with ideal properties to be used as epitopes in following imprinting processes. This concept has been applied, for example, in the development of MIPs to serve as allosteric modulators of acetylcholinesterase activity [71]. These presented excellent functionality, with a particular formulation increasing the enzyme activity by 47 × and recovering its activity from 11% (following exposure to an inhibitor) to 73%. In a similar application, this procedure was repurposed for efficient proteomic mapping of cancer cell surfaces, aiming to identify accessible diagnostic markers and potential therapeutic targets [12]. In the tested cancer cell Lines, this allowed the classification of 438 proteins, with 283 considered to be transmembrane or extracellular proteins, creating a “proteomic fingerprint” of the surface of each cell type. Additionally, three previously unknown epitopes of EGFR were generated and used to successfully produce EINPs for selective recognition of this cancer marker.

Another method for epitope identification leverages sequence similarity to design versatile EINPs capable of recognizing multiple peptides or glycans within the same family. This has been applied to create imprinted “nanotags” for labelling certain glycosylation patterns of interest [73]. Using a clever combination of imprinting strategies, Li et al. engineered improved surface-enhanced Raman scattering (SERS) platforms for precise diagnosis of hepatocellular carcinoma based on the relative glycosylation of the biomarker alpha fetoprotein (AFP). First, the C-terminal epitope of AFP was used to prepare an imprinted substrate that captured the protein from samples. To detect the total level of captured protein, EINPs against the N-terminal epitope and decorated with Raman reporter PATP (4-aminothiophenol) were used. Then, EINPs imprinted with the A2G2S2 glycan structure and tagged with 4-NTP (4-nitrothiophenol) were used to quantify the proportion of AFP modified with different glycans containing this specific structure, which is associated with malignancy. This dual detection strategy enabled simultaneous quantification of both total and A2G2S2-glycosylated AFP, allowing for accurate ratiometric analysis of clinical samples.

#### Monomers and Small Molecules as Epitopes

In a more simplified approach, smaller molecules such as a single amino acid have also been proven to be effective templates for targeting specific proteins. Given that a single monomer cannot be as idiotypic as a whole sequence, this strategy compromises selectivity and affinity for a faster and less costly development with a more practical implementation. For instance, fluorenylmethyloxycarbonyl (Fmoc)-protected phosphorylated single amino acids have been successfully investigated as template epitopes for the targeting of phosphorylated peptides, focusing particularly on phosphotyrosine (pY) [74, 75], but also demonstrating applicability for phosphoserine [76] and phosphohistidine [77] residues. Phosphorylation is a key post-translational modification that regulates various biological processes, including cell growth, division, and apoptosis. Abnormal phosphorylation patterns are often associated with diseases such as cancer, diabetes, and neurodegenerative disorders. Thus, identifying phosphorylation patterns can prove an important step to identify certain diseases or predict responses to particular therapies, helping in the construction of personalized interventions. This approach has been successfully validated against the benchmark methods for phosphorylated amino acid enrichment, involving either titanium oxide (TiO_2_) or anti-pY antibodies [78]. Moreover, besides functioning only as an alternative to traditional methods, it has also been combined with TiO_2_ to selectively enrich protein extracts from cancer cells in phosphopeptides before mass spectrometry analysis [74], suggesting its potential to improve phosphoproteomics research. As an alternative to Fmoc-pY, other groups have instead explored the use of phenylphosphonic acid (PPA) as a dummy template for identification of pY residues, owing to the extremely similar molecular structures of PPA and pY [79, 80]. This method takes advantage of a lower cost template molecule to obtain increasingly impressive results in terms of affinity, adsorption capacity and selectivity, stretching the boundaries of the epitope concept.

Besides amino acids, monosaccharides like glucuronic acid and N-acetylneuraminic acid have proven effective as epitope templates. Nanoparticles imprinted with these sugars were successfully used to recognize and fluorescently label specific glycosylation sites in macromolecules like (proteo)glycans, for example, hyaluronic acid, considering the abundance of these specific sugar monomers in their structures [81–83]. Notably, these nanoparticles exhibited no cross-reactivity with other common monosaccharides found at the terminal ends of proteoglycans or glycoconjugates on cell surfaces, proving their remarkable selectivity despite the simplicity of this approach. These pioneering saccharide EINPs thus demonstrate tremendous potential for the development of new theragnostic tools, since altered glycosylation levels or particular glycan patterns are indicative of pathological conditions such as infections or cancer malignancy [82]. Additionally, other studies have also tested the combination of a single monosaccharide with a single amino acid (mannose and tryptophan, respectively) to imprint microspheres, showing a satisfactory recognition ability for the glycopeptide antibodies telavancin and teicoplanin [84, 85]. It remains to be seen if this strategy can improve recognition of more complex macromolecules, such as cell surface glycoproteins, compared to saccharide imprinting only.

Despite the encouraging advances in empirical epitope identification methods, they still show significant performance limitations. For proteins with a real interest as biomarkers or therapeutic targets, which inevitably have prohibitive costs, methods based on their direct and disposable usage in digestion protocols present a notable obstacle. On the other hand, approaches relying on single monomers or small molecules have limited scope of target proteins, relying on specific structural properties. This invites the development of more efficient, rational approaches that can be universally used to identify proteotypic epitopes for any kind of target, widening the range of potential applications.

### Rational Strategies for Epitope Identification

The shortcomings presented by empirical epitope selection prompted the development of new *rational approaches*. These methods aim to maximize the affinity and selectivity of MIPs by carefully designing the imprinted template so that its structure and properties best mimic the epitope in the native protein. These strategies have been progressively improved and standardized into workflows based on bioinformatic tools, allowing the comparative analysis and alignment of protein sequences, 3D visualization of protein structures, search for naturally occurring epitopes, and simulation of molecular interactions, with continuously improving outcomes.

#### Learning from Nature—Immunogenic and Functional Epitopes

An attractive strategy for rational epitope selection draws inspiration from the original concept of epitope as a fraction of a protein that is recognized by an antibody. Thus, EINPs can be conceived as synthetic equivalents of antibodies and be imprinted against already known immunogenic sequences. Bioinformatic platforms such as the Immune Epitope Database (IEDB, accessible at www.iedb.org) [86] are valuable resources with ever increasing information that allow researchers to easily retrieve previously verified and tested immunogenic epitopes for specific proteins of interest, including linear and discontinuous (structured) peptides, as well as non-peptidic epitopes. Moreover, this platform also offers a host of Tools (IEDB Analysis Resource, located at tools.iedb.org) for epitope mapping, prediction, and analysis based on a.a. sequences as well as 3D structures, including several MHC prediction methods, Epitope Conservancy Analysis [87], Homology Mapping [88], or ElliPro structural prediction [89]. These tools can aid in the design of new epitopes analogous to natural ones, which is particularly useful when natural epitope sequences are not compatible with MI methods or for proteins that have not yet been extensively documented. In this context, Lu et al. developed a sensing platform for HIV detection by imprinting a sequence corresponding to a major immunodominant region of HIV-1 glycoprotein (gp)41 (a.a. 579–613), attaining an affinity comparable to preexisting mAbs (*K*_*D*_ = 3.17 nM) and a detection limit similar to mAb-based enzyme-linked immunosorbent assays (ELISA) [90].

Similarly, the use of structures or sequences identified as functional parts of a protein, i.e., responsible for interacting with other proteins (receptor/ligand, enzyme/substrate, binding partner, etc.) has been demonstrated to be an efficient way to select epitopes for MI [91]. Resources such as STRING [92], BioGRID [93], or IntAct [94] can be leveraged to identify other proteins interacting with the target and their experimentally determined interaction sites. For proteins with a well-known 3D structure (via PDB or AlphaFold [95]), analyzing it for potential binding grooves, pockets, or interaction interfaces is another path to identifying potential imprinting candidates. Docking simulations performed with the aid of HADDOCK [96] or ClusPro [97] can further help to observe how the proteins dynamically interact and identify key residues involved.

**Fig. 4:**
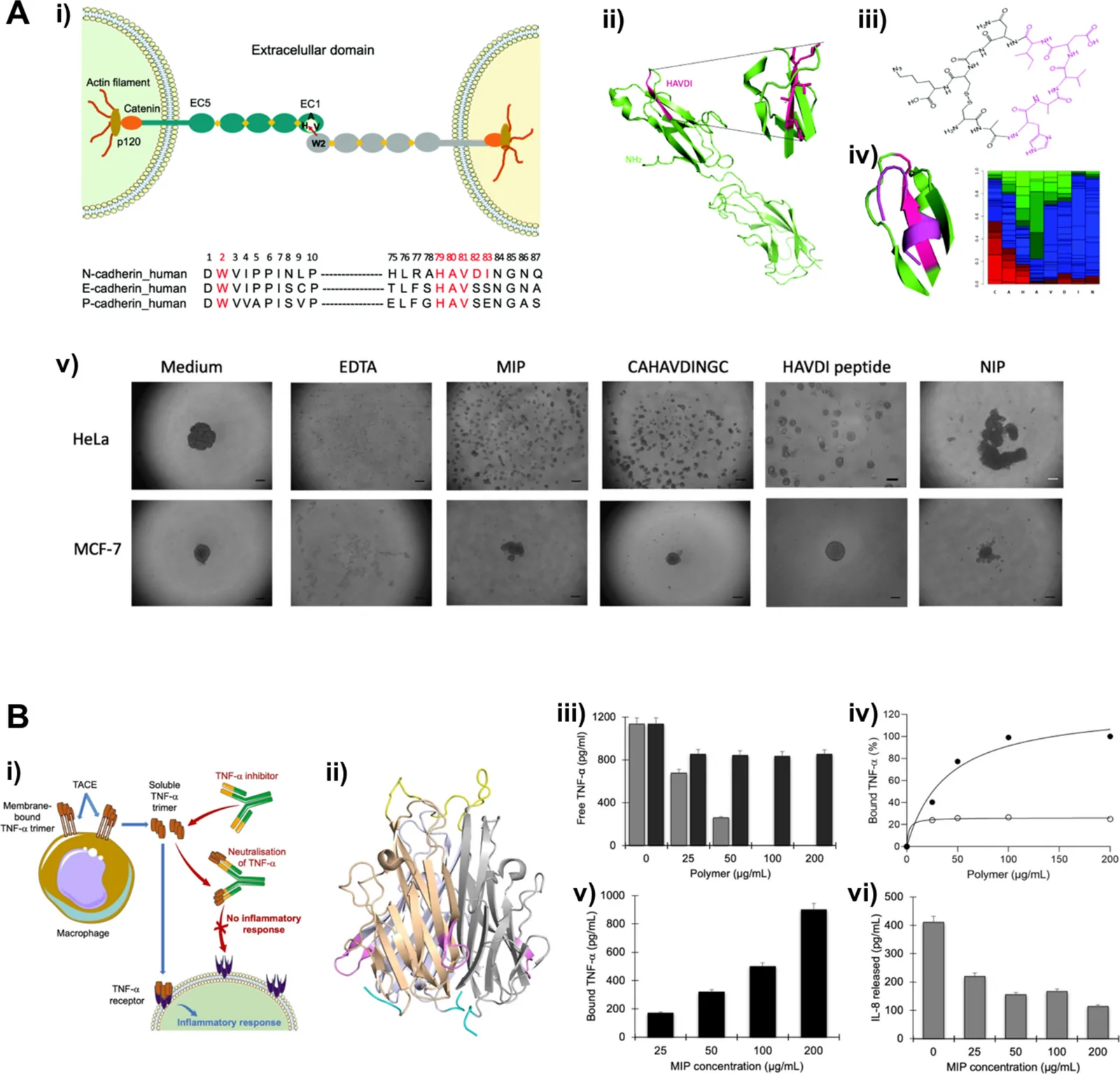
Rational strategies for epitope selection. **A** Imprinting of structured epitopes derived from protein-interfacing regions. (i) (above) Schematic representation of trans dimerization of cadherins. The five ectodomains (EC1 to EC5) are represented as grey and green ovals. Adhesion occurs by the insertion of W2 from one cadherin into the hydrophobic pocket lined by HAV in the EC1 of the adjacent cadherin. (below) Multiple sequence alignment showing the amino acids (red) in human E-, N- and P-cadherins, involved in cell adhesion. Alignment performed with EMBL-EBI. (ii) Crystal structure of murine N-cadherin domains EC 1–2 (PDB entry: 2QVI) with highlighted HAVDI sequence. (iii) Chemical structure of constructed cyclic template peptide NH_2_-CAHAVDINGCK(N_3_)-COOH. (iv) (Left) Superposition of the predicted 3D structure of the template peptide (flanked by 2 Cys) (violet), onto the native protein epitope. (Right) PEP-FOLD2 prediction of secondary structure probabilities associated to the template amino acids residues. Green: extended, blue: coil, red: helix. (v) EINPs are able to disrupt the aggregation of N-cadherin-expressing HeLa cells, but not of MCF-7 cells which express E-cadherin. Microscopy images display cell aggregation when incubated with 4 mM ethylenediaminetetraacetic acid (EDTA), 20 μg mL^−1^ EINPs (MIP), 100 nM template peptide (CAHAVDINGC), 100 nM HAVDI Linear peptide and 20 μg mL^−1^ non-imprinted nanoparticles (NIP), in growth medium. Scale bars: 250 μm. Adapted with permission [100]. Copyright 2022, Royal Society of Chemistry. **B** Template selection by a systematized bioinformatic workflow [118]. (i) Scheme depicting tumor necrosis factor (TNF)-α inhibitor preventing TNF-α from binding to its receptor, thereby suppressing inflammatory signaling. (ii) Crystal structure of homotrimeric TNF-α (PDB entry: 1TNF) showing the most promising epitopes, at the N-terminus (blue) and two internal epitopes (pink and yellow). The epitope in yellow, TPEGAEA, was used to prepare EINPs. iii) Blocking of cell-secreted TNF-α by EINPs (grey) and control NIPs (black). (iv) Binding isotherms for EINPs (black circles) and NIPs (white circles). (v) Sequestration of TNF-α by increasing concentrations of EINPs. (vi) subsequent downregulation of interleukin (IL)−8 release by THP-1 differentiated macrophages. Adapted with permission [119]. Copyright 2023, Wiley

Alternatively, for proteins where structural data is limited or unavailable, predictive methods like PSIVER [98] can be applied to determine residues likely to be involved in protein–protein interactions based on sequence data. In an example of this concept, Medina-Rangel et al. developed EINPs targeting conserved a.a. sequences (DWVIPPI [99] and AHAVDING [100]) in the region responsible for the N-terminal trans-dimerization of specific types of cadherins that mediates cell–cell adhesion in particular tumors. These nanoparticles were used to immunostain different cancer cell lines (HaCaT, MCF-7, and HeLa), with the staining intensity correlating with the reported levels and types of cadherins in each cell Line, thus demonstrating the specificity of these EINPs. Moreover, they were able to inhibit HeLa cell adhesion and migration, as well as disrupting 3D tumor spheroids, revealing tremendous promise for anti-cancer therapeutic applications (Fig. 4A).

Similarly, Batista et al. engineered EINPs as plastic antibodies for the SARS-CoV-2 spike protein [101]. The authors analyzed the crystallographic structure of the receptor-binding domain (RBD) of this protein when bound to angiotensin-converting enzyme 2 (ACE2) from human cells, the entry point for SARS-CoV-2. This resulted in the identification of the sequence FNCYFPLQSYGFQPTNG (F486–G502) from the viral protein. Despite its length, this peptide presents 10 contact moieties with ACE2 during interaction, providing a suitable candidate for imprinting, as demonstrated by the imprinting factor of 4.1 obtained by the prepared MIPs when interacting with the full spike protein. Interestingly, Bognár et al. followed the same rationale and narrowed the target sequence down to the nonapeptide GFNCYFPLQ (G485–Q493), attaining a *K*_*D*_ of *ca*. 20 nM for the RBD region and down to the sub-picomolar range for viral-like particles, showing an impressive binding ability [102].

#### An Additional Mimetic Layer—Structured Epitopes

While most studies on epitope imprinting use linear peptides, the dominant mechanism of recognition in Nature is based on interactions with secondary and tertiary structures of proteins and thus, discontinuous sequences [103]. Therefore, an innovative strategy to recreate native protein structures in synthetic epitopes is the design of conformational peptides. In an example of this method, Xu et al. used molecular modelling to draw a cyclic peptide partially containing a natural epitope of the target HIV gp41 (SWSNKS) to develop EINPs as plastic antibodies to prevent HIV infection [91]. In this case, the template was prepared with cysteine residues at both ends (CGSWSNKSC), allowing its oriented immobilization and adoption of a circular conformation similar to the native sequence. The affinity of resulting EINPs for the target HIV gp41 was 85.4 nM, and cross-reactivity was below 2% for other tested proteins, corroborating the success of this approach.

Going one step further, Zhang et al. leveraged the natural α-helical structure of the apamin toxin peptide, using it as a scaffold to recreate the exposed N-terminal α-helix of hyaluronan-binding protein 1, also known as p32, a surface receptor known to be overexpressed in several cancer types [104]. To construct the required template, residues in apamin (^10^L, ^12^A, ^13^R, ^14^R, ^16^Q, ^17^Q, and ^18^H) were replaced with the topologically equivalent ones from the target p32 (^79^D, ^81^A, ^82^F, ^83^ V, ^85^F, ^86^L, and ^87^S). They have demonstrated that the preservation of three scaffold amino acids was critical to impart the helical structure to the new sequence: ^9^A, which exhibits the highest α-helix propensity, and two cysteines for disulfide bond stabilization. EINPs imprinted with this structured peptide could efficiently bind the target protein, recognizing p32-positive tumor cells, and therefore successfully apply targeted photodynamic therapy in vivo in mice.

In another study dedicated to targeted cancer therapy, Hashemi-Moghaddan et al. systematically applied a set of bioinformatic tools as described above in the optimization of structured epitope design [105]. First, IEDB was used to perform epitope mapping, followed by PyMOL molecular model viewer [106] and ElliPro for structural prediction, resulting in four candidate conformational epitopes for human epidermal growth factor receptor 2 (HER2), an overexpressed receptor in various cancers. Finally, Protein–Protein Basic Local Alignment Search Tool (BLASTP) [107] was applied to determine the candidate with the highest correspondence to the target HER2, simultaneously showing the lowest predictable cross-reactivity with other undesired proteins. EINPs against this template were applied in vivo, demonstrating the capability of selectively delivering loaded doxorubicin to tumors overexpressing this receptor while diminishing drug spreading to healthy tissues. Remarkably, treated mice presented more than double the cumulative survival compared to control groups, demonstrating the potential of this strategy for treatment of HER2-positive cancers.

Our group has similarly made use of the previous approaches, combining them to design a conformational epitope for transforming growth factor-β3 (TGF-β3) [22]. We started by retrieving the protein sequence from RCSB PDB and using PyMOL for its 3D visualization in order to find surface-exposed regions that did not interfere with receptor binding, followed by BLASTP to rank the identified candidate peptides. Intriguingly, the N-terminal α-helix was ranked as the most promising candidate. Therefore, based on the previous work, we reconstructed this epitope using apamin as a molecular scaffold and predicted the resulting structure using PEP-FOLD 3 de novo peptide structure prediction server (accessible at https://bioserv.rpbs.univ-paris-diderot.fr/services/PEP-FOLD3/) [108], confirming the similar spatial distribution of a.a. side chains. The prepared EINPs showed selective affinity for the full TGF-β3 protein even at low concentrations within a mixture of proteins with similar isoelectric points and other isoforms of TGF-β.

#### Towards a Universal Systematic Selection Method

As discussed above, selecting an adequate MI epitope can be performed in a number of different ways and considering several key criteria, ranging from the accessibility of the targeted region to peptide length, physicochemical characteristics (e.g., solubility, charge) or its structure (or lack thereof). This complex and critical step has been increasingly standardized by different groups into a more unified protocol, facilitating future research in biomacromolecule MI.

Pioneered by the Bossi group [109], a streamlined process termed *fingerprint analysis* has been optimized for rationally choosing such an idiotypic epitope [11], according to the following steps: (a) select target protein sequence and obtain it from database (NCBI Protein [available at https://www.ncbi.nlm.nih.gov/protein/] [110], UniProt, RCSB PDB, etc.); (b) cut it in silico into peptides by choosing a suitable cutting agent (e.g., trypsin) using ExPASy PeptideCutter (available at https://web.expasy.org/peptide_cutter/) [111]; as mentioned above, peptides beyond the 8–16 a.a. length limits should be discarded; (c) peptides of adequate length are aligned to the source database using BLASTP; the most promising epitope is the one which best matches the parental protein (highest S score), while simultaneously presenting the lowest *E* value (i.e. the number of distinct alignments that occur in the database by chance). This simple but efficient workflow can further be combined with additional platforms for 3D visualization, surface functionality assessment, or secondary structure prediction, such as PEP-FOLD3, Mol*, or PyMOL [22]. Additionally, it is important to assess basic characteristics of the resulting candidate epitopes, e.g., charge or solubility, to ensure its suitability for the imprinting procedure, for which tools like ExPASy ProtParam (accessible at https://web.expasy.org/protparam/) or Innovagen PepCalc.com (at https://pepcalc.com/) can be used.

This process has shown to be particularly useful for generating MIPs to identify peptides resulting from proteolytic digestion of proteins, for example, for mass spectroscopy analysis [112]. Cenci et al. have focused on developing EINP-based platforms for cardiac troponin T detection, allowing early diagnosis of heart failure [113]. Our group has also shown that this technique is efficient for generating EINPs capable of binding the full target protein, in this case, TGF-β3 [22, 114]. These nanoparticles could be applied as artificial growth factor receptors, potentiating its paracrine signaling and allowing precise control over stem cell differentiation without external growth factor supplementation, thus showing potential for making tissue engineering constructs more cost-efficient and widely available. By adapting this workflow, Bartold et al. developed an EINP-based sensor for detection of matrix metalloproteinase-1 (MMP-1), a biomarker associated with idiopathic pulmonary fibrosis, a progressive and fatal lung disease with limited diagnostic options [115]. The authors started by screening the protein sequence to identify four candidate peptides based on parameters such as their length, stability, hydrophilicity, and location on the protein surface. Next, the structural stability of the selected peptides was modeled using computational chemistry techniques. Semi-empirical PM7 calculations [116] (implemented in the MOPAC2016 software, available at https://OpenMOPAC.net/), were used to optimize epitope conformations and identify the lowest-energy configurations. Then, the authors calculated the Gibbs free energy change (ΔG) for the formation of complexes between epitopes and different functional monomer mixtures using density functional theory methods in Gaussian 16 software [117]. Based on these analyses, two epitopes (MIAHDFPGIGHK and HGYPKDIYSS) were chosen due to their ability to form stable pre-polymerization complexes with functional monomers and their advantageous structural properties. This approach ensured that the chosen epitopes were both chemically stable and structurally suitable for selective recognition by MIPs.

More recently, Mier et al. introduced an improved workflow for rational epitope synthesis with promising results [52, 118]. Briefly, the crystallographic structure of the protein of interest (from UniProtKB, for example), is examined to choose flexible loops or coil fragments that are surface-exposed to solvents, with sequences 8–16 a.a. long moving to the next stage. Then, from the table of “amino acid modifications” in UniProtKB, fragments where post-translational modifications and disulfide bonds take place are discarded, since disulfide bonds between the chosen epitope and a different protein region would implicate this epitope in a native conformation that cannot be reproduced by the free peptide epitope. Additionally, glycosylation sites can be resolved using the servers NetOGlyc 4.0 and NetNGlyc 1.0 (accessible at http://www.cbs.dtu.dk/services/NetOGlyc and http://www.cbs.dtu.dk/services/NetNGlyc/, respectively). PEP-FOLD3 is then used to analyze the sequences of the resulting peptide(s), predicting their secondary structure, with the goal of being as close as possible to the native protein fragment. This visualization can further be aided by Mol* or PyMol, as previously mentioned. Lastly, either the “Peptide search” function in the UniProtKB database (available at http://www.uniprot.org/peptidesearch) or BLASTP can be applied to verify if the peptide sequence is also present in unrelated proteins. The candidate peptide(s) is therefore considered to be the one with the most similar structure to its corresponding portion in the target protein while simultaneously presenting a sequence unique to that protein.

This approach was successfully demonstrated in the development of EINPs for Hepatitis A Virus Cell Receptor-1 (HAVCR-1), the point of entry for hepatitis A virus and the filoviruses Ebolavirus and Marburgvirus, which is also known to be overexpressed in various cancer types [118]. The resulting nanogels attained an IC_50_ value of 14 nM for the full HAVCR-1 protein, showing good potential for antiviral therapies by blocking the binding site of the protein, thereby preventing filoviruses from infecting host cells, or even as anticancer theranostics agents. The same workflow was applied to select an epitope of tumor necrosis factor (TNF)-α, a potent pro-inflammatory cytokine that plays key roles in diseases such as rheumatoid and psoriatic arthritis, multiple sclerosis and Crohn's disease [119]. EINPs against the optimal epitope (TPEGAEA, a.a. 181–187) could neutralize TNF-α in the supernatant of human THP-1 macrophages, leading to the downregulation of pro-inflammatory cytokine secretion by these immune cells (Fig. 4 B). This strategy thus holds considerable promise for generating more cost-effective and accessible, fully synthetic TNF-α inhibitors, aiming to treat a variety of inflammatory diseases and thereby improve patient quality of life.

As explored throughout this section, bioinformatic methods have been increasingly applied, optimized, and systematized to facilitate and improve the selection of the best epitope for MI [120, 121]. The fast-expanding knowledge on protein structures made possible by AI technologies, particularly Google DeepMind’s AlphaFold [122], will certainly power these efforts even further in coming years, allowing a better analysis of a wide array of proteins that have been difficult targets due to their unresolved structures. Evolving this type of deep learning systems more specifically for epitope prediction and design is likely to be a breakthrough point in the near future, fast-tracking this critical step of EINP development, and therefore making the advantages of this technology more widely available.

## Present Limitations and Challenges

Although epitope imprinting has evolved considerably and is increasingly featured in emerging strategies for disease diagnosis and targeted therapies, no MIP-/EINP-based products have yet been approved for clinical use. This highlights the need to address various challenges to improve their translational potential. The developments in epitope selection strategies mentioned above have contributed to pushing the specificity of EINPs to levels comparable to or even surpassing those of monoclonal antibodies, the current golden standard in targeted therapeutics (Table 1). An adequate template choice and performance evaluation help to ensure that prepared MIPs can identify the target (glyco)protein with high affinity and selectivity. In this regard, it is essential to avoid the pitfall of targeting potentially inaccessible epitopes, such as those buried within the native conformation of the protein or masked by post-translational modifications [19].

**Table 1:** Summary of the main design elements and key performance indicators for EINPs in the surveyed literature

Target/biomarker	Epitope selection method	Epitope(s)	Monomers, crosslinker(s)	Synthesis technique	K_D_ (nM)*	Reference
Hyaluronan	Empirical, terminal, disease-associated	Glucuronic acid (GlcA)	AAB, MAm, polymerizable rhodamine B (PolyFluor 570), EGDMA	Precipitation polymerization	IF = 3.2	[44]
EGFR	Empirical, terminal	SLNITSLGLRSLKEISDG (a.a. 418–435), doxorubicin	NIPAm, TBAm, APMAm, AA, FAm, MBAm	SPI	3.6	[51]
CD59	Empirical, terminal	YNCPNPTADCK	DMAEMA, NIPAm, TBAm, BAC	Surface imprinting on nanoparticles via free-radical polymerization	IF = 3.5	[53]
GPNMB	Empirical, terminal	KRFHDVLGN	APTES, BnTES, IBTES, UPTES, TEOS	ROSIC	IF = 12.3	[54]
B2M	Empirical, terminal	KIVKWDRDM	APTES, BnTES, IBTES, UPTES, TEOS	ROSIC	16.3	[54]
HER2	Empirical, terminal	TQVCTGTDM	APTES, BnTES, IBTES, UPTES, TEOS	ROSIC	11.8	[54]
GPNMB	Empirical, terminal	KRFHDVLGN	APTES, BnTES, IBTES, UPTES, TEOS	ROSIC	40.2	[55]
SIRPα	Empirical, terminal	ELKVTQPEKSV	APTES, IBTES, UPTES, TEOS	BOSIC	62.2 (epitope)	[56]
PD-L1	Empirical, terminal	FTITAPKDLYVV	APTES, BnTES, IBTES, UPTES, TEOS	BACOSI	IF = 5.1	[57]
VEGF165, VEGF121	Empirical, terminal, receptor binding	APMAEGGGQNHH	APTES, IBTES, UPTES, TEOS	BOSIC	1.92 (VEGF_165_), 1.89 (VEGF_121_)	[58]
REG1B	Empirical, physicochemical properties	NEDRETWVDADLY	EVAL	Surface imprinting on nanoparticles	IF = 2.6	[61]
REG1B	Advanced experimental selection, physicochemical properties, structural features	KSWDTGSPSSANAGYCAS	EVAL	Phase inversion surface imprinting	1.73 pg/ml	[64]
ProGRP	Advanced experimental selection, target digestion	NLLGLIEAK	DMAEMA, EAMA, MAA, HEMA, 4-VPy, TFMAA, TFU, DVB, EGDMA	Thermal polymerization followed by crushing and sieving	3400	[69]
ProGRP	Advanced experimental selection, target digestion	NLLGLIEAK	EAMA, DVB	Surface imprinting on nanoparticles	7100	[70]
HER2	Advanced experimental selection, target digestion	N-glycans from the extracellular domain of HER2	APTES, TEOS	BACOSI	IF = 8.02	[41]
EGFR	Advanced experimental selection, snapshot imprinting	KLFGTSGQK LTQLGTFEDHFLSLQR NLQEILHGAVR	NIPAm, TBAm, APMAm, AA, MBAm	SPI	47.1 16.8 31.9	[12]
EGFR	Advanced experimental selection, snapshot imprinting	KLFGTSGQK GMNYLEDR GVLGSGAFGTVYK NLQEILHGAVR MHLPSPTDSNFYR LTQLGTFEDHFLSLQR	AA, APMA, FAm, NIPAm, TBAm, MBAm	SPI	47.1 0.2 22.2 31.9 11 16.8	[124]
AFP	Advanced experimental selection, shared sequence associated with disease state	A2G2S2 glycan structure	APTES, BnTES, IBTES, UPTES, TEOS	BACOSI	NA	[73]
Phosphotyrosine-containing peptides	Small molecule with structural similarity	Phenylphosphonic acid	UPTES, TEOS	Epitope imprinting on mesoporous silica nanoparticles	IF = 4.59	[80]
Sialic-acid overexpressing glycoproteins	Overexpressed monomer	Sialic acid (N-acetyl neuraminic acid—NANA)	4-vinylbenzeneboronic acid, 2-aminoethyl-methacrylate hydrochloride, 2-(−3-(4-nitrobenzo[c][1, 2, 5] oxadiazo-7-yl)ureido) ethylmethacrylate, EGDMA	ternary complex-based surface imprinting	1.7 × 10^5^ (free SA) 208 to 278 (cell lines)	[43]
Aberrant glycosylation sites	Overexpressed monomer	GlcA, NANA	AB, MAm, HEMA, MBAm, EGDMA	Surface imprinting on nanoparticles	140 000 (GlcA) 20 000 (NANA)	[81, 82]
Hyaluronan	Overexpressed monomer	GlcA	AB, NIPAm, MBAm	SPI	800 (GlcA)	[83]
HIV glycoprotein 41	Rational selection, structural feature, receptor binding	CGSWSNKSC	NIPAm, TFMAA, TBAm, PAm, MBAm	SPI	79.6 (epitope)	[91]
E- and N-cadherins	Rational selection, functional relevance	DWVIPPI	AB, NIPAm, PAm, TBAm, MBAm	SPI	IC_50_ = 2.5 nM (E-cadherin) IC_50_ = 20 nM (N-cadherin)	[99]
E- and N-cadherins	Rational selection, functional relevance	AHAVDING	AB, NIPAm, TBAm, MBAm	SPI	IC_50_ = 41 nM (E-cadherin) IC_50_ = 25 nM (N-cadherin)	[100]
SARS-CoV-2 spike protein	Rational selection, receptor binding, structurally relevant	FNCYFPLQSYGFQPTNG	APTES, PTES, UPTES, TEOS	Oriented surface imprinting on nanoparticles	IF = 4.1	[101, 102]
HER2	Rational selection, bioinformatic workflow, conformational	CPLHNOEKCSKPCARV	Dopamine	Oriented surface imprinting on nanoparticles	NA	[105]
Cardiac troponin I	Rational selection, bioinformatic workflow	NIDALGSMEGR	AAm, HEMA, MAA, TBAm, MBAm	Precipitation polymerization	1.2	[113]
HAVCR-1	Rational selection, bioinformatic workflow, conformational	EHRGWFND	AB, PAm, NIPAm, TBAm, MBAm	SPI	IC_50_ = 14 nM	[118]
TNFα	Rational selection, bioinformatic workflow, conformational	TPEGAEA	AB, NIPAm, TBAm, MBAm	SPI	NA	[119]
ERα	Rational selection, functional relevance, immunogenic	SHSLQKYYITGEAEGFPATV	AA, APMAm, NIPAm, TBAm, FMA, MBAm	SPI	10.8–14.7	[123]

*Dissociation constant (*K*_*D*_) is noted whenever it is available in the cited reference. Imprinting factor (IF) is provided whenever *K*_*D*_ could not be found

Target/biomarker: *AFP* alpha-fetoprotein, *B2M* beta2 microglobulin, *EGFR* epidermal growth factor receptor, *ERα* estrogen receptor alpha, *GPNMB* glycoprotein NMB, *HAVCR-1* Hepatitis A Virus Cellular Receptor 1, *HER2* human epidermal growth factor receptor-2, *PD-L1* programmed death-ligand 1, *ProGRP* progastrin-releasing peptide, *REG1B* regenerating protein 1B, *TNFα* tumor necrosis factor alpha

Monomers/crosslinkers: *4-VPy* 4-vinylpyridine, *AA* acrylic acid, *AAB* (*N*-acrylamido)-benzamidine, *AB* (4-acrylamidophenyl)(amino)methaniminium acetate, *AAm* acrylamide, *APMAm N*-(3-aminopropyl)methacrylamide hydrochloride, *APTES* 3-aminopropyltriethoxysilane, *BAC* N,N′-diacrylylcystamine, *BnTES* benzyltriethoxysilane, *DMAEMA* dimethylaminoethyl methacrylate, *DVB* divinylbenzene, *EAMA N*-(2-aminoethyl) methacrylamide hydrochloride, *EGDMA* ethylene glycol dimethacrylate, *EVAL* poly(ethylene-co-vinyl alcohol), *FAm N*-fluoresceinylacrylamide, *FMA* fluorescein-*O*-methacrylate, *HEMA* 2-hydroxyethyl methacrylate, *IBTES* isobutyltriethoxysilane, *MAA* methacrylic acid, *MAm* methacrylamide, *MBAm N,N′*-methylene-bis-acrylamide, *NIPAm N*-isopropylacrylamide, *PAm N*-phenylacrylamide, *PTES* phenyltriethoxysilane, *TEOS* tetraethyl orthosilicate, *TFMAA* trifluoromethacrylic acid, *TFU N*−3,5-bis(trifluoromethyl)-phenyl-N′−4-vinylphenylurea, *UPTES* 3-ureidopropyltriethoxysilane

Synthesis techniques: *BACOSI* boronate-affinity-controllable oriented surface imprinting, *BOSIC* boronate affinity–anchored epitope-oriented surface imprinting and cladding, *ROSIC* reverse microemulsion-confined epitope-oriented surface imprinting and cladding, *SPI* solid phase imprinting

A critical hurdle is the validation of recognition capability in biologically relevant conditions. In such environments, EINPs are exposed to complex matrices containing countless molecules with structures and physicochemical properties close to those of the target. This can result in nonspecific adsorption, particularly of abundant serum proteins like albumin, and competitive binding with off-target species. Besides the epitope selection, monomer choice, synthesis method, and nanoparticle design all critically influence EINP performance. Significant progress in these areas has also been made recently. For instance, employing mixtures of functional monomers tailored to the chemical nature of the template residues can enhance binding affinity by optimizing interactions between the target and the imprinted cavities. Both acrylate/acrylamide-based and silane-based monomer mixtures have demonstrated promising outcomes [55, 73, 123, 124], although there is a need for more representative in vivo assays to further validate these approaches.

In vivo administration raises additional concerns regarding nanoparticle biodistribution, clearance pathways, and long-term biocompatibility, challenges faced broadly by nanomedicine platforms, which are equally relevant for EINPs [125, 126]. However, very few studies have directly focused on these aspects. For example, Kassem et al. report that EINPs prepared by solid phase imprinting and administered in mice either intravenously or orally were able to reach every organ tested, including the central nervous system, being excreted through both fecal and urinary routes [127]. Additionally, the study noted dose-dependent toxicity as well as signs of inflammatory and immune reactions when EINPs were administered orally. While these results established a relatively safe dose threshold, they underscore the importance of incorporating nanoparticle design and engineering strategies for modulation of pharmacokinetics. These include both materials design (e.g., steric repulsion, size tuning, endosomal escape) and bioengineering (e.g., reticuloendothelial system blockade, microenvironmental reprogramming, permeability enhancement) approaches. In parallel, development of fully biodegradable MIPs based on natural polymers is another emerging strategy that can help to overcome some of these barriers, namely, long-term toxicity and in vivo persistence [128].

Other major barriers to clinical application of new MIP-based medical products are production scaleup and alignment with regulatory standards. While epitope imprinting offers a theoretical edge in cost efficiency over traditional techniques using large biomacromolecules, bioreactors, or live animals, this has not yet translated into off-the-shelf products. This could be related to the lack of scalable, reproducible production technologies with high yields and attaining narrow, well-defined size distributions, allowing full control over EINP properties [129]. Nevertheless, some studies are finally attempting to bridge this gap, focusing on more automated, innovative reactor systems that allow a continuous production routine more compatible with industrial standards [130, 131]. Combining these advances with increasingly powerful computational approaches and machine learning/artificial intelligence could significantly accelerate the development of clinically viable EINPs.

Finally, progress toward regulatory approval will also depend on standardization efforts and compliance with established quality management frameworks. This includes adherence to good manufacturing practices (GMP), implementation of robust quality control protocols, and alignment with international regulatory standards (e.g., ISO—International Organization for Standardization). Without consistent protocols for characterization and batch validation, clinical translation remains difficult. Regulatory bodies such as the US Food and Drug Administration (FDA) and the European Medicines Agency (EMA) will require comprehensive data on product reproducibility, safety, and efficacy demonstration before MIP- or EINP-based systems can enter the clinical pipeline [126].

## Conclusions and Perspectives

Epitope molecular imprinting technology has rapidly advanced, addressing many challenges associated with imprinting entire macromolecules like proteins in polymeric matrices. Compared to biological alternatives—such as antibodies or aptamers—EINPs offer greater versatility, lower production costs, scalability, and long-term stability. These properties position them as strong candidates for theranostics applications. Additionally, the synthetic nature of EINPs is well-aligned with the growing ethical pressure to reduce animal use in R&D, a priority gaining momentum among policymakers and the public.

Recent innovations have propelled epitope imprinting toward high-value molecular targets, including circulating biomarkers, cancer cell surface proteins, and growth factors. These advancements pave the way for real-world applications in diagnostics, regenerative medicine, and targeted therapies. The rise of bioinformatics and molecular modeling tools together with increased computational power accelerates epitope selection and polymer optimization, streamlining MIP development while reducing their costs. As the accessibility of this technology improves, more research groups globally are contributing to the field, expanding its application scope in a wide range of the biomedical areas such as drug delivery [51, 132], tissue engineering [22, 114], drug-free anti-cancer therapies [100, 119], and precision diagnostics [133, 134].

Despite this progress, significant challenges remain. Continued development of automated, large-scale production systems will be essential for the democratization of EINP access, allowing present and future companies to quickly upscale the tailor-made synthesis capabilities of these materials for commercial purposes. The appearance of new companies using this technology (e.g., MIP Diagnostics Ltd, SEMOREX), particularly leveraging the advantages offered by the solid phase imprinting method, is an encouraging sign that a turning point is being reached for MIPs. We foresee a future where clinicians and researchers can swiftly request EINPs tailored for specific proteins or diseases, receiving customized materials within weeks. Standardized protocols for design, testing, and quality control will be essential to support this transition from lab to clinic.

These firm advances in recent years provide confidence in our vision of EINPs as real *plastic antibodies*, enabling novel solutions across biomedicine and clinical care.

## Data Availability

The authors confirm that the data supporting the findings of the study are available within the article.
